# Supplementary material for: Splice-Junction-Based Mapping of Alternative Isoforms in the Human Proteome
Source: Cell Rep. Author manuscript; Available in PMC 2020 Jan 15. (PMC6961840; doi:10.1016/j.celrep.2019.11.026)
Supplement: 2 [file NIHMS1546469-supplement-2.zip › DF1/PXD006675/LeftCoronaryArtery/LeftCoronaryArtery_7_SNRNP25_AAQAGAMVVQDPLLCDLPIQVTLEEVNSQ.pdf]

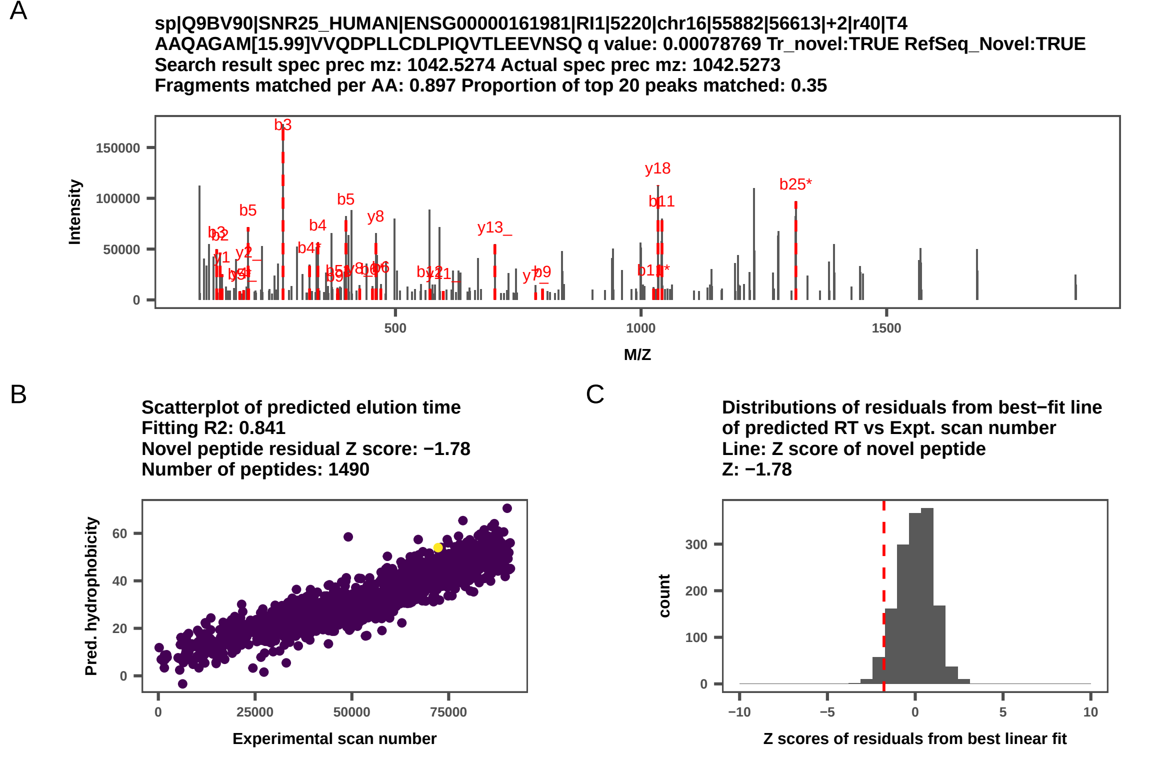
A
sp|Q9BV90|SNR25_HUMAN|ENSG00000161981|RI1|5220|chr16|55882|56613|+2|r40|T4
AAQAGAM[15.99]VVQDPLLCDLPIQVTLEEVNSQ q value: 0.00078769 Tr_novel:TRUE RefSeq_Novel:TRUE
Search result spec prec mz: 1042.5274 Actual spec prec mz: 1042.5273
Fragments matched per AA: 0.897 Proportion of top 20 peaks matched: 0.35
Intensity
150000
100000
50000
0
b3
y18
b25*
b11
b5
b5
b4
y8
y13_
M/Z
500
1000
1500
B
Scatterplot of predicted elution time
Fitting R2: 0.841
Novel peptide residual Z score: −1.78
Number of peptides: 1490
Pred. hydrophobicity
60
40
20
0
0
25000
50000
75000
Experimental scan number
C
Distributions of residuals from best−fit line
of predicted RT vs Expt. scan number
Line: Z score of novel peptide
Z: −1.78
count
300
200
100
0
−10
−5
0
5
10
Z scores of residuals from best linear fit
